# Prolonged Slow Expiration Technique and Gastroesophageal Reflux in Infants Under the Age of 1 Year

**DOI:** 10.3389/fped.2021.722452

**Published:** 2021-09-08

**Authors:** Laure Lievens, Yvan Vandenplas, Sylvie Vanlaethem, Filip Van Ginderdeuren

**Affiliations:** ^1^Rehabilitation Research, Department of Physiotherapy, Human Physiology and Anatomy, Faculty of Physical Education and Physiotherapy, Vrije Universiteit Brussel, Brussels, Belgium; ^2^KidZ Health Castle, Universitair Ziekenhuis Brussel, Vrije Universiteit Brussel, Brussels, Belgium; ^3^Physiotherapy Department, Universitair Ziekenhuis Brussel, Brussels, Belgium

**Keywords:** Prolonged Slow Expiration, airway clearance techniques, impedance, pH monitoring, infant, gastroesophageal reflux, respiratory physiotherapy

## Abstract

**Background:** The Prolonged Slow Expiration Technique (PSE) is an airway clearance technique (ACT) carried out in newborn children with bronchial obstruction and hypersecretion to clear away the mucus from the respiratory tract. Evidence about the effect of PSE on gastroesophageal reflux (GER) is currently lacking in the literature. This study aimed to evaluate the influence of PSE on GER in infants under the age of 1 year.

**Methods:** Infants were observed using multichannel intraluminal impedance-pH monitoring (MII-pH) over 24 h. During monitoring, the participants were treated with one 20 min intervention of PSE in supine position, 2 h after feeding. In this controlled trial with intra-subject design, the number of reflux episodes (REs) during PSE were compared to 20 min before and after PSE.

**Results:** Fifty infants younger than 1 year were screened of whom 22 had a pathological GER. For the entire group, no significant difference was seen in the total number of REs between before, during, or after the PSE treatment (*P* = 0.76). No significant difference in total REs was found between the three measuring points (*P* = 0.59) in the group of infants with an abnormal MII-pH (*n* = 22).

**Conclusion:** PSE does not cause a significant difference in REs in infants younger than 1 year.

**Registration number:** NCT03341585.

## Introduction

Airway clearance techniques (ACT's) are widely used to facilitate mucociliary clearance in the respiratory tract. Expiration Lente Prolongée (ELPr) is the French term for the Prolonged Slow Expiration (PSE) Technique developed by Guy Postiaux, a Belgian physiotherapist and used in infants with bronchial obstruction and hypersecretion (1). The technique involves slow bimanual thoraco-abdominal pressure applied at the end of the expiratory phase. This pressure brings the patient to the expiratory reserve volume (ERV) (2, 3). The obtained lung deflation and difference between pleural and mouth pressure help to facilitate secretion clearance (4). Postiaux et al. showed that to reduce symptoms of bronchial obstruction in mild acute bronchiolitis, PSE and provoked cough is a safe method (2). Nogueira et al. concluded that PSE is a reliable chest physical therapy technique with reproducible results between therapists (5). Conesa-Segura et al. showed the PSE reduces Acute Bronchiolitis Severity Scale scores and reduced the length of hospital stay without detecting any adverse events (3).

Certain ACT's can exacerbate gastroesophageal reflux (GER) (6). GER is defined in the Pediatric Gastroesophageal Reflux Clinical Practice Guidelines as “the passage of gastric contents into the esophagus or oropharynx, with or without regurgitation, and/or vomiting” (7). This normal physiological process lasts <3 min, occurs most often after a meal, and is for the most part associated with transient relaxations of the lower esophageal sphincter (LES) (8, 9). GER becomes GER disease (GERD) when it increases in intensity and frequency and leads secondarily to troublesome symptoms that affect daily functioning (7, 10). GERD can be associated with extra-esophageal symptoms such as coughing, wheezing, choking, and may impair pulmonary function through reflex bronchospasm and micro-aspiration (10, 11). The range of symptoms attributable to GERD broadly overlaps with the normal behavior of infants, like regurgitation, excessive crying, and irritability (12). Of all reflux episodes (REs) in infants, up to 90% are non-acid (13).

To measure GER, 24-h esophageal multichannel intraluminal impedance monitoring in combination with pH-metry (MII-pH) is currently the most sensitive tool as it measures both acid and non-acid REs (14). Advantages of MII-pH are the ability to detect the nature (air-liquid), direction (swallowing-reflux), and pH (acid pH <4, non-acid pH >4) of the content in the esophagus (14). The relation of both acid as non-acid REs to respiratory problems must be taken into account. Non-acid REs in infants were more associated with persistent respiratory symptoms than acid REs and mainly non-acid REs precede cough (15–17). Gastric content with low ionic substances (such as air) results in high impedance values and gastric content with high ionic substances (saliva, reflux of food) results in low impedance levels. The height and duration of REs can be determined by measuring the distance and time of impedance changes (18).

To our knowledge, only one study examined the influence of PSE on GER (19). Reychler et al. showed that PSE in a seated position can cause GER (19). Limitations of this study were the large differences in ages between subjects (between 0 and 18 years) and the use of conventional pH-metry which measures only acid GER (19).

This study aimed to assess the influence of PSE in supine position on GER in infants younger than 1 year. The primary outcome was the number of REs. The number of REs during the intervention was compared to the number of REs during baseline (control).

## Materials and Methods

### Patients

Each child younger than 1 year, referred to the hospital to confirm a clinically suspected GERD diagnosis with 24-h MII-pH was included. After informing their parents about this controlled trial, participation was proposed. Those parents who participated provided written informed consent. Since reflux treatment could bias our results (20), infants who were treated with medication against GER were excluded. If proton pump inhibitors (PPIs) were given, infants were not excluded if the PPIs were stopped more than 1 week prior to the investigation. The second exclusion criterion was a gestational age of <37 weeks. The investigation was approved by the UZ Brussel ethics committee (B.U.N.143201734076) and registered at ClinicalTrials.gov (NCT03341585).

### Materials

Each 24-h MII-pH was performed using a Sandhill Scientific MII-pH monitoring system (Denver, CO, USA) and an appropriate infant MII-pH catheter with seven impedance sensors and one distal pH-sensor (calibrated in pH 4.0 and pH 7.0 buffers). The position of the catheter was adjusted by chest X-ray so that the insertion of the pH-sensor was at the third vertebral body above the diaphragmatic angle (21). During the three measuring points, a computer program (Sleuth Zephr Impedance/pH Reflux Monitoring System, Sandhill Scientific Inc., 2011) calculated the number of REs. An experienced pediatric gastroenterologist (YV) read out the full MII-pH.

An impedance RE was defined as a retrograde drop in impedance by more than 50% of baseline in at least two distal impedance sensors (22). The REs were as acid if the pH was lower than 4.0 for more than 5 s and as non-acid if the pH was above 4.0 for more than 5 s. If the pH was acidic for more than 7% of the intervention time or if the number of reflux episodes was more than 100 over a 24-h period, the MII-pH was considered abnormal (7, 18).

### Intervention

All patients were placed in supine position and treated with the PSE technique, for 20 min, by two experienced and trained physiotherapists (FV, SV). PSE was carried out as follows: the hypothenar region of one hand, placed under the sternal notch of the infant's chest, applied gentle pressure at the end of the expiratory phase in the cranio-caudal direction and at the same time the hypothenar region of the other hand, below the umbilicus of the infant's abdomen, moved in the opposite direction (3). The pressure was kept for two or three breathing cycles. The technique is repeated between 30 and 33 times, with a rest time between applications of about 5 or 10 spontaneous breaths. At the end of the last inspiratory phase, coughing is triggered by applying brief tracheal pressure above the sternal notch (3).

The intervention started 2 h post-prandial to exclude any influence of feeding on GER. The number of REs were observed before (control), during, and after treatment with the patient in the same supine position. REs were calculated by a computer program without human interference and MII-pH recordings were analyzed by an experienced pediatric gastroenterologist (YV).

### Statistical Analysis

To provide normal values for infants younger than 1 year, our group performed in a previous study an interim analysis of 15 infants implementing MII-PH (21). Over sixty-one 20 min periods (2 h post-prandial), REs were measured, resulting in a mean of 0.98 (*SD* = 0.66) REs for each infant (21). These results were used during power calculation, which estimated that 50 subjects would be required to detect a 50% change in the number of REs with a power of 95% at the 5% significance level (21). Shapiro-Wilk and Kolmogorov-Smirnov Goodness of Fit tests showed that the data were not normally distributed. To compare differences in REs between baseline, the intervention period, and 20 min after the intervention, Friedman's Two-way Analysis of Variance Test was used. Statistical significance is considered at *P* < 0.05.

## Results

### Baseline Characteristics of the Participants

**Figure 1:**
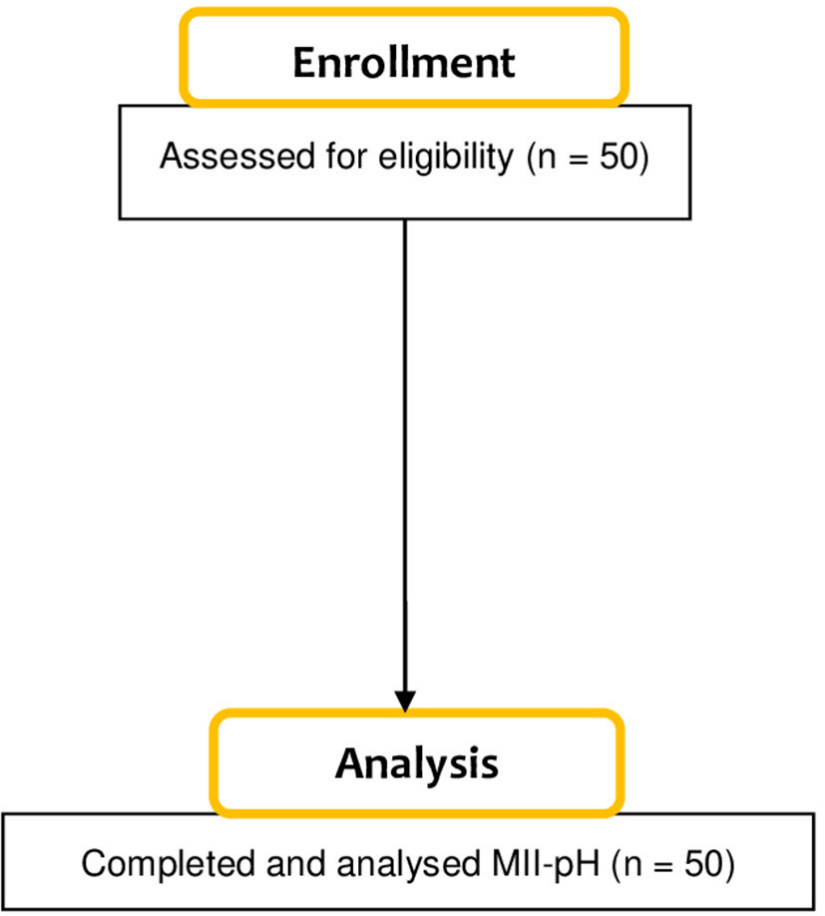
Recruitment of study participants.

To reach the desired power, fifty infants younger than 1 year were screened (Figure 1). Infants were their own controls. Demographics of the included participants are presented in Table 1. Eighteen infants (7 boys and 11 girls) were referred because of troublesome regurgitation and vomiting, 17 infants (11 boys and 6 girls) because of a chronic cough and/or wheezing, 12 infants (9 boys and 3 girls) because of inconsolable crying, and 3 infants (1 boy and 2 girls) because of suspicion of brief, resolved, unexplained events. MII-pH monitoring was performed for a mean duration of 22 h and 50 min. Upon the MII-pH results, 22 infants showed an abnormal MII-pH and received a reflux diagnosis.

**Table 1:** Patients demographics.

Sex (male/female)	28/22
Median age in days (range)	133 (15–354)
Reflux diagnosis (yes/no)	22/28
Reason for referral	
Regurgitation/vomiting (%)	18 (36)
Cough/wheezing (%)	17 (34)
Crying (%)	12 (24)
Brief, resolved, unexplained events (%)	3(6)

### MII-pH Monitoring

#### Reflux Episodes Before Treatment (Baseline), 2 H Post-prandial

Thirty-two infants (64%) had any REs during the 20-min baseline period just before treatment. Sixteen infants (32%) showed acid REs and 14 infants (28%) non-acid REs. Both acid and non-acid REs were seen in two infants (4%). Eighteen infants (36%) showed no pre-treatment REs. In total, 60 REs were registered. Thirty-two (53%) of these REs were acid, 28 REs (47%) were non-acid (Tables 2, 3).

**Table 2:** Results of the MII-pH monitoring: differences between pre-PSE, PSE, and post-PSE.

			**Total**	**Pre-PSE**	**PSE**	**Post-PSE**	***P*-value**
All patients	N° infants		50	50	50	50	
	N° infants with REs			32 (64%)	29 (58%)	31 (62%)	0.82^*^
	N° REs		203	60 (30%)	74 (36%)	69 (34%)	0.76^**^
		Medians (IQR 25–75%)		1 (0–2)	1 (0–2)	1 (0–2)	0.76^**^
All patients with abnormal MII-pH	N° infants		22	22 (44%)	22 (44%)	22 (44%)	
	N° infants with REs			15 (68%)	17 (77%)	17 (77%)	0.73^*^
	N° REs		113	28 (25%)	42 (37%)	43 (38%)	0.59^**^
		Medians (IQR 25–75%)		1 (0–2)	1.5 (0.75–2.25)	1.5 (0.75–3)	0.59^**^
All patients with normal MII-pH	N° infants		28	28 (56%)	28 (56%)	28 (56%)	
	N° infants with REs			17 (61%)	12 (42%)	14 (50%)	0.40^*^
	N° REs		90	32 (36%)	32 (36%)	26 (29%)	0.82^**^
		Medians (IQR 25–75%)		1 (0–2)	0 (0–2)	0.5 (0–1.75)	0.82^**^

*PSE, Prolonged Slow Expiration; N°, number; REs, reflux episodes. Statistics:*

* *Crosstabs, Chi-square, P = 0.05*.

** *Friedman test, P = 0.05*.

**Table 3:** Results of the MII-pH monitoring with *post-hoc* analysis.

		**Total**	**Pre-PSE**	**PSE**	**Post-PSE**	***P*-value**
All patients	N° infants	50	50	50	50	
	N° infants with REs		32 (64%)	29 (58%)	31 (62%)	0.82^*^
	N° REs	203	60 (30%)	74 (36%)	69 (34%)	0.76^**^
	N° acid REs		32 (53%)	32 (43%)	21 (30%)	0.22^**^
	N° non-acid REs		28 (47%)	42 (57%)	48 (70%)	0.69^**^
All patients with abnormal MII-pH	N° infants	22	22 (44%)	22 (44%)	22 (44%)	
	N° infants with REs		15 (68%)	17 (77%)	17 (77%)	0.73^*^
	N° REs	113	28 (25%)	42 (37%)	43 (38%)	0.59^**^
	N° acid REs		12 (43%)	15 (36%)	9 (21%)	0.38^**^
	N° non-acid REs		16 (57%)	27 (64%)	34 (79%)	0.74^**^
All patients with normal MII-pH	N° infants	28	28 (56%)	28 (56%)	28 (56%)	
	N° infants with REs		17 (61%)	12 (42%)	14 (50%)	0.40^*^
	N° REs	90	32 (36%)	32 (36%)	26 (29%)	0.82^**^
	N° acid REs		20 (63%)	17 (53%)	12 (46%)	0.24^**^
	N° non-acid REs		12 (38%)	15 (47%)	14 (54%)	0.83^**^
All patients referred for regurgitation/vomiting	N° infants	18	18 (36%)	18 (36%)	18 (36%)	
	N° infants with REs		16 (89%)	13 (72%)	9 (50%)	0.04^*^
	N° REs	89	27 (30%)	35 (39%)	27 (30%)	0.26^**^
	N° acid REs		18 (67%)	22 (63%)	12 (44%)	0.10^**^
	N° non-acid REs		9 (33%)	13 (37%)	15 (56%)	0.62^**^
All patients referred for cough/ wheezing	N° infants	17	17 (34%)	17 (34%)	17 (34%)	
	N° infants with REs		11 (65%)	9 (53%)	12 (71%)	0.56^*^
	N° REs	69	26 (38%)	22 (32%)	21 (30%)	0.87^**^
	N° acid REs		11 (42%)	6 (27%)	5 (24%)	0.58^**^
	N° non-acid REs		15 (58%)	16 (73%)	16 (76%)	0.98^**^
All patients referred for crying	N° infants	12	12 (24%)	12 (24%)	12 (24%)	
	N° infants with REs		5 (42%)	6 (50%)	7 (58%)	0.72^*^
	N° REs	35	7 (20%)	12 (34%)	16 (46%)	0.10^**^
	N° acid REs		3 (43%)	4 (33%)	4 (25%)	0.87^**^
	N° non-acid REs		4 (57%)	8 (37%)	12 (75%)	0.31^**^
All patients referred for BRUE	N° infants	3	3 (6%)	3 (6%)	3 (6%)	
	N° infants with REs		0 (0%)	1 (33%)	3 (100%)	0.04^*^
	N° REs	10	0 (0%)	5 (50%)	5 (50%)	0.20^**^
	N° acid REs		0 (0%)	0 (0%)	0 (0%)	
	N° non-acid REs		0 (0%)	5 (100%)	5 (100%)	0.20^**^

*PSE, Prolonged Slow Expiration; N°, number; REs, reflux episodes. Statistics:*

* *Crosstabs, chi-square, P = 0.05*.

** *Friedman test, P = 0.05*.

#### Reflux Episodes During Prolonged Slow Expiration

During the 20-min lasting intervention of PSE, 74 REs in 29 infants (58%) were detected. Thirty-two REs (43%) were acid and 42 REs (57%) were non-acid. Acid REs were found in 12 (24%) infants, non-acid in11 (22%) children. Six infants (12%) showed both acid as non-acid REs. Twenty-one infants (42%) showed no REs during the intervention (Tables 2, 3).

#### Reflux Episodes After the Prolonged Slow Expiration Treatment

In the 20-min period following treatment, 69 REs in 31 infants (62%) were seen. Twenty-one (30%) of these REs were acid, 48 (70%) were non-acid. Ten (20%) infants showed acid REs, non-acid was seen in 18 (36%) infants. Three infants (6%) showed both. Nineteen (38%) children showed no REs following treatment (Tables 2, 3).

#### Comparison of Reflux Episodes Between Before, During, and After the Prolonged Slow Expiration Treatment

For the entire group, no statistically significant difference in the total number of REs between before, during, or after the PSE treatment (*P* = 0.76) was found. In the group of infants with a 24-h abnormal MII-pH result (*n* = 22), also no significant difference in the total REs was detected between the three measuring points (*P* = 0.59). Same results were found for the group with normal MII-pH (*n* = 28, *P* = 0.82; Table 2).

This study was underpowered for detecting differences according to the result of the 24-h MII-pH recording. However, *post-hoc* analysis was carried out (Table 3). In the total group (*P* = 0.22) and in the groups with abnormal MII-pH (*P* = 0.38) and normal MII-pH (*P* = 0.24) no significant differences were found for acid REs. For non-acid REs, in the total group (*P* = 0.69) and in the separate groups with abnormal MII-pH (*P* = 0.74) and normal MII-pH (*P* = 0.83) also no significant differences were objectified.

**Figure 2:**
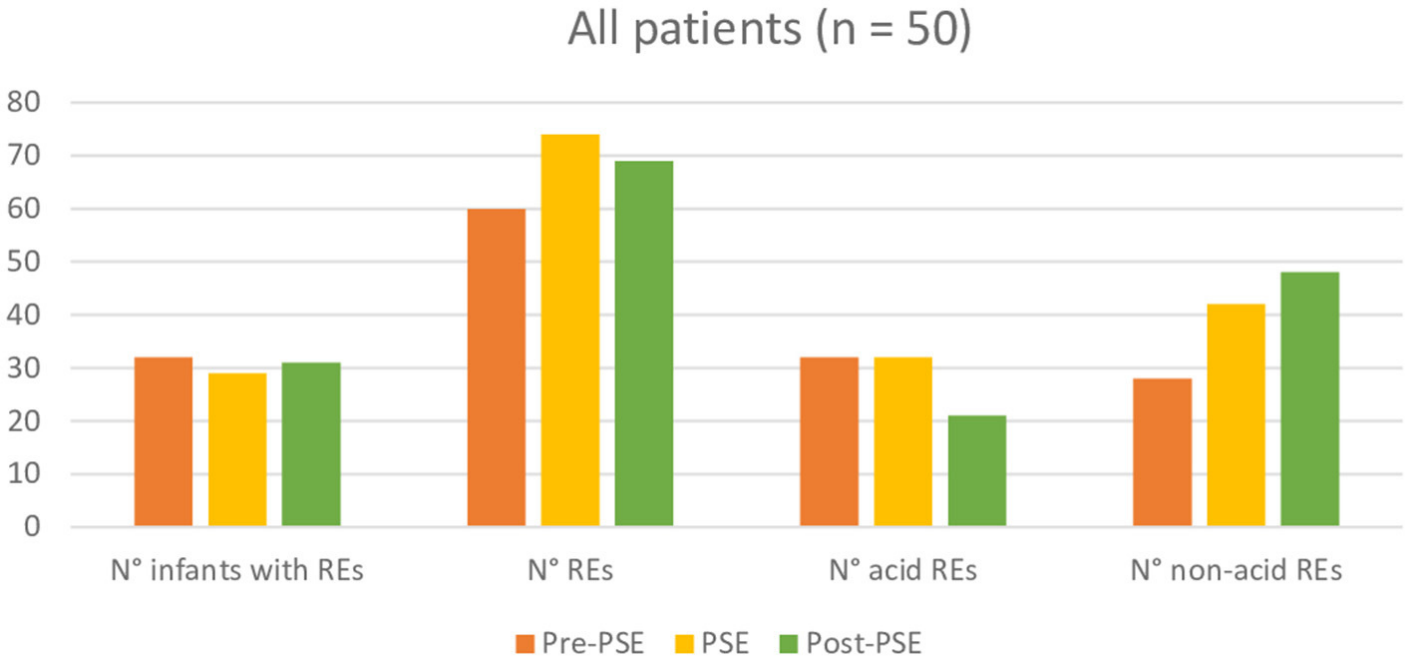
Comparison of reflux episodes between before, during, and after the Prolonged Slow Expiration (PSE) treatment in the entire group.

**Figure 3:**
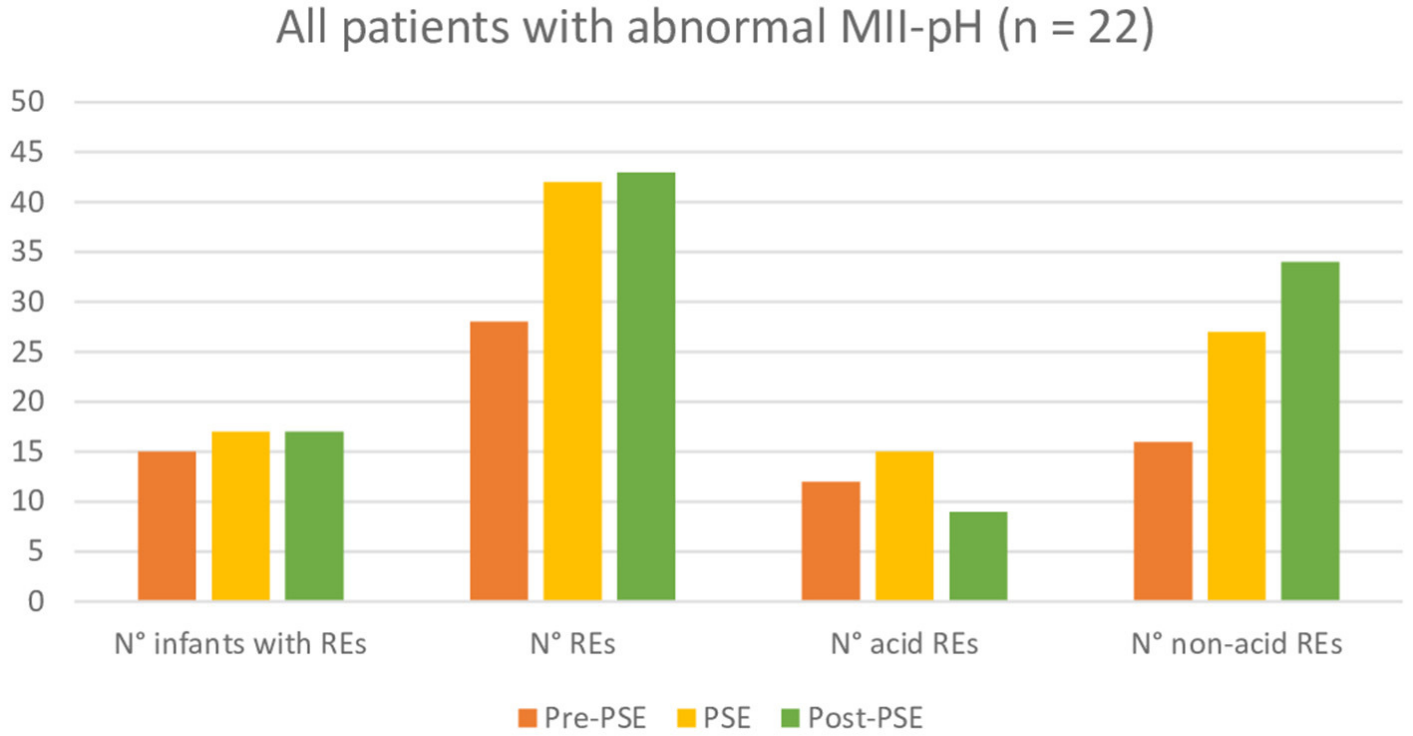
Comparison of reflux episodes between before, during, and after the Prolonged Slow Expiration (PSE) treatment in the group with abnormal MII-pH.

**Figure 4:**
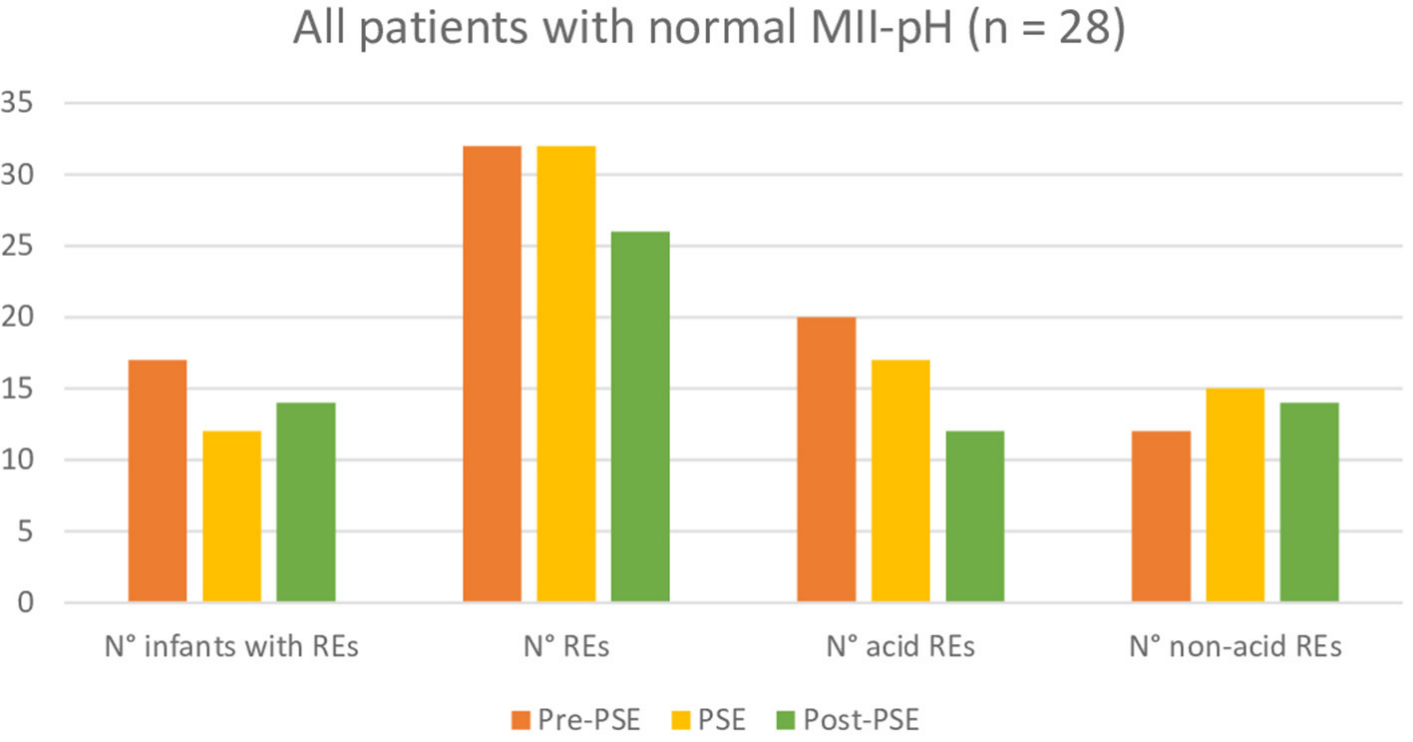
Comparison of reflux episodes between before, during, and after the Prolonged Slow Expiration (PSE) treatment in the group with normal MII-pH.

Further *post-hoc* analysis, indicated that participants regardless of their reason for referral have no significant difference in total, acid, or non-acid number of REs between the three measuring points. More detailed and additional information can be found in Figures 2–4.

## Discussion

The results of this study revealed that the Prolonged Slow Expiration Technique, regardless of the indication or result of the 24 h MII-pH monitoring, does not cause a difference in reflux episodes in infants younger than 1 year. The effect of PSE on GER has not yet been thoroughly investigated. Only one group, Reychler et al. proved that PSE in a seated position can lead to REs in children, regardless of the presence of GERD (19). In contrast to our study conventional pH-metry was used, no pre or post-treatment measurement was registered, the range in age of the included children was between 0 and 18 years, and the participants were placed in a seated position.

Different positions do influence GER. Previous investigations about the influence of chest physiotherapy on GER mainly focused on head-down tilt position possibly in combination with chest clapping, vibrations, thoracic compression, and percussion (6). It is known that the use of gravity-assisted positions is not recommended (6). Head-elevated position is a position decreasing the number of GER episodes, head-down position is a risk factor inducing GER (23, 24). A prone-elevated positioning in a harness is more effective in decreasing GER than in a seated positioning (25). A right lateral and supine position increases the number REs while prone and left lateral position decreases the number of REs (26). Iwakiri et al. found that LES pressure in a supine position was significantly higher than those in a seated position in both patients with reflux esophagitis and healthy subjects (27). Moreover during PSE thoracoabdominal pressure was carried out. It is known that a greater thoracoabdominal pressure gradient could lead to more GER (28). However, we can conclude that the PSE treatment in a supine position in children under the age of 1 year is a safe, non-reflux-inducing technique.

In addition, in the study of Reychler et al. (19) the age range was large. As we know that infants have a much higher incidence of regurgitation than older children, there is a bias risk in this study. To prevent this bias, our study focused on infants under the age of 12 months, as regurgitation decreases from birth, and tends to disappear mostly by 12 months of age.

In a previous study, our group investigated the influence of assisted autogenic drainage (AAD) on GER (29). Esophageal pH monitoring over 24 h was used to detect the RE and the patients were treated in a supine position. Despite the supine position we proved that AAD does not cause more acid GER in infants younger than 1 year.

To investigate the influence of respiratory physiotherapy on GER, studies carried out with MII-pH are still limited (22, 30, 31). The main advantage of MII-pH in comparison with conventional pH-metry is the ability to detect both acids as non-acid REs instead of only acid REs. In several studies, the importance of acid RE in its relation to respiratory problems has been investigated. Rosen et al. demonstrated that non-acid REs in infants were more associated with persistent respiratory symptoms than acid REs (15). Reflux, and mainly non-acid REs, were shown to precede cough (16, 17). In our study no significant difference was found in acid or non-acid REs between groups.

No pre-or post-treatment measurement was registered in the study of Reychler et al. (19). This allows our study to be more accurate as we can compare the intervention period with a standardized baseline. Reychler et al. (19) applied each technique over 5 min, with an interval of 5 min between both (19). This is in contrast to our study, where the technique was applied over 20 min. This 20-min period is much more in line with the application in clinical practice.

Modern ACT's successfully replace older techniques such as clapping and postural drainage. These techniques have a negative influence on GER. IPV (30), PSE, and AAD whether or not combined with bouncing (29) are the three ACT's proven to be safe and efficient in the clearance of mucus in babies without exacerbating GER.

Some limitations of our study are the inclusion of patients from a single center which may affect the generalizability of the results and the inclusion of patients with motility disorders and significant esophagitis where the number of REs may be underestimated during MII-pH as a result of low baseline impedance values.

## Conclusion

Prolonged slow expiration, regardless of the indication or result of the 24 h MII-pH monitoring, does not cause a significant difference in reflux episodes in infants under the age of 1 year.

## Data Availability Statement

The raw data supporting the conclusions of this article will be made available by the authors, without undue reservation.

## Ethics Statement

The studies involving human participants were reviewed and approved by B.U.N.143201734076. Written informed consent to participate in this study was provided by the participants' legal guardian/next of kin.

## Author Contributions

LL: literature search, analysis of data, and manuscript preparation. YV: study design, analyzes of measurements, and review of the manuscript. SV: study design and data collection. FG: data collection, study design, and review of manuscript. All authors contributed to the article and approved the submitted version.

## Conflict of Interest

The authors declare that the research was conducted in the absence of any commercial or financial relationships that could be construed as a potential conflict of interest.

## Publisher's Note

All claims expressed in this article are solely those of the authors and do not necessarily represent those of their affiliated organizations, or those of the publisher, the editors and the reviewers. Any product that may be evaluated in this article, or claim that may be made by its manufacturer, is not guaranteed or endorsed by the publisher.
